# Multivariate Analysis of the Survival Rates and Risk Factors of One-Piece Zirconia Implants Supporting Single Crowns or Fixed Dental Prostheses: A Retrospective Cohort Study with Follow-Up Periods of up to 8 Years

**DOI:** 10.3390/dj14050282

**Published:** 2026-05-09

**Authors:** Jorge Cortés-Bretón Brinkmann, Santiago Bazal-Bonelli, María Jesús Suárez, Cristina Meniz-García, Cristina Madrigal Martìnez-Pereda, Juan López-Quiles

**Affiliations:** 1 Department of Dental Clinical Specialties, Faculty of Dentistry, Complutense University of Madrid, Plaza Ramón y Cajas S/N, 28040 Madrid, Spain; cmenizga@ucm.es (C.M.-G.); cmadriga@ucm.es (C.M.M.-P.); jlopezq@ucm.es (J.L.-Q.); 2 Surgical and Implant Therapies in the Oral Cavity Research Group, Complutense University of Madrid, 28040 Madrid, Spain; 3 Department of Conservative Dentistry and Buccofacial Prosthesis, Faculty of Dentistry, Complutense University of Madrid, 28040 Madrid, Spain; mjsuarez@odon.ucm.es

**Keywords:** dental implants, zirconium oxide, guided bone regeneration, smoking

## Abstract

**Background/Objectives**: Titanium implants remain the gold standard in implant dentistry. However, growing interest in metal-free alternatives has led to increased use of zirconia implants. Despite encouraging short-term outcomes, evidence regarding the medium- to long-term survival of one-piece zirconia implants (O-PZIs) and associated risk factors remains limited. The aim of this retrospective cohort study was to evaluate the survival of O-PZIs over follow-up periods of up to 8 years and to explore variables potentially associated with implant failure. **Methods**: This retrospective observational cohort study was conducted at a private dental clinic (Madrid, Spain). A total of 307 O-PZIs placed in 196 patients between 2017 and 2021 were analyzed. Implant survival was assessed using Kaplan–Meier analysis, while associations between clinical variables and implant failure were explored using chi-square tests and multivariate Cox regression models (*p* < 0.05). The mean follow-up period was 61.37 ± 2.25 months. **Results**: After a mean follow-up of 61.37 ± 2.25 months (range: 39–96 months), 42 failures were recorded, resulting in a cumulative survival rate of 86.32% (CI 95%: 79.28–92.96%). Most failures (64.29%) occurred before prosthetic loading. Kaplan–Meier analysis revealed significantly lower survival for tapered implants (*p* < 0.001) and among smokers (*p* < 0.001). Multivariate analysis indicated that only simultaneous guided bone regeneration (GBR) was independently associated with implant failure (Exp(B) = 3.191; 95% CI: 1.299–7.840; *p* = 0.011). However, this association should be interpreted with caution due to the retrospective design, potential confounding, limited number of events, and lack of adjustment for clustering at the patient level. The discrepancies observed between statistical methods highlight the importance of time-to-event analyses in implant research. **Conclusions**: Within the limitations of this study, O-PZIs demonstrated acceptable medium- to long-term survival. Simultaneous GBR may be associated with increased risk of failure. However, these findings should be considered exploratory. Further prospective studies are required to confirm these results and to better define risk factors in ceramic implant therapy.

## 1. Introduction

Titanium and its alloys remain the most widely used materials for dental implant manufacture due to their excellent biocompatibility, favorable mechanical properties, and well-documented long-term survival rates. Extensive clinical evidence supports their predictability, making them the gold standard in implant dentistry. These materials have significantly improved the clinical outcomes of tooth replacement therapies [1,2,3].

Nevertheless, despite their overall success, certain biological and esthetic limitations have been reported, prompting interest in alternative materials [4,5]. Possible adverse biological reactions have been described, with one systematic review reporting high prevalence rates of peri-implant mucositis (43%) and peri-implantitis (22%) associated with titanium implants [6,7]. In this context, Wachi et al. argue that worsening of mucositis may be the result of titanium ions released by corrosion, which will evolve into peri-implantitis leading to bone resorption [8]. Although these complications are multifactorial and not exclusively material-dependent, they have led to ongoing research into alternative biomaterials [9].

Another problem is patient hypersensitivity to titanium. Several articles state that some patients—albeit very few—exhibit clinical signs of allergy to titanium and/or their traces [10]. Additionally, titanium’s dark gray color can compromise esthetic outcomes. This is a more frequent problem for patients with thin periodontal biotypes in the anterior region, as the metal may become visible through the peri-implant mucosa [11,12].

These problems have driven the development and clinical uptake of alternative materials, with ceramics becoming more widely used due to their superior esthetic properties [13]. In particular, yttria-stabilized tetragonal zirconia polycrystal (Y-TZP) has gained prominence due to its fracture resistance, which is well within clinically acceptable limits, as shown by in vitro studies [14]. Alumina-toughened zirconia (ATZ) implants are also recommended for their enhanced mechanical stability compared with Y-TZP [15].

At present, a wide range of zirconia implant brands and designs are available. One-piece and, more recently, two-piece ceramic implants are increasingly being used with promising results. A recent systematic review (SR) concludes that O-PZIs appear to be a reliable option for restoring missing teeth, with an implant survival rate of 94.5% and a success rate of 92% after follow-up periods of at least 3 years [5].

It is important to distinguish between ‘implant survival’ (defined as the implant remaining in situ regardless of complications) and ‘implant success,’ which demands stricter biological and prosthetic criteria [16]. While short- to medium-term outcomes of zirconia implants are promising, long-term data—particularly beyond five years—remain limited. Moreover, given the inherent limitations of one-piece implants, there is no clear evidence of the risk factors that can determine the success or failure of this type of ceramic implant. Therefore, the primary objective of this study was to establish the survival rate of one-piece implants with follow-up periods of up to 8 years. The secondary objective was to determine which potential risk factors are critical to compromising implant survival and to provide clinically relevant data to inform treatment planning and patient management.

## 2. Materials and Methods

### 2.1. Study Design and Approval

This retrospective observational cohort study aimed to assess clinical outcomes and to explore variables potentially associated with O-PZI failure. All patients attended a private dental clinic (Madrid, Spain), receiving at least one implant placed between January 2017 and January 2021; a total of 221 patients received 346 O-PZIs. However, 25 patients missed follow-up appointments and/or disappeared at some point during the therapeutic process and were thus withdrawn from the study. Eighteen patients from other European countries (predominantly the UK and Germany) attended the clinic solely for O-PZI placement and did not return for subsequent periodic follow-up. Additionally, five patients relocated to another city, and two patients passed away during the follow-up period. Consequently, data from 307 O-PZIs in 196 patients were analyzed.

Patients were contacted retrospectively and, for the purposes of the study, gave their consent for the use of their medical histories and details of the clinical procedures undergone. All procedures met the ethical standards of the institutional and/or national research committee for research involving human subjects and the 1964 Helsinki Declaration and subsequent amendments. The study was conducted following the STROBE (Strengthening the Reporting of Observational Studies in Epidemiology) guidelines [17].

The study protocol was assessed and approved by the Research Ethics Committee at the San Carlos Hospital of Madrid, Spain, in 17 November 2025 (Registration Code No. 25/7611-E).

### 2.2. Population

All patients were adults (≥18 years) of either male or female sex, classified as ASA physical status I or II, presenting one or more missing teeth requiring implant therapy. All consented to both surgical and follow-up procedures and received subsequent restoration with single crowns (SCs) or fixed dental prostheses (FDPs).

Exclusion criteria comprised pregnancy or lactation, inability to attend follow-up visits, systemic diseases or treatments contraindicating implant placement (including infectious diseases, uncontrolled diabetes, recent cardiovascular events, neurodegenerative disorders, autoimmune conditions, and use of immunosuppressants or anticoagulants, among others).

### 2.3. Data Collection

A case history was created for each participant. One researcher (S.B.B.) reviewed patients’ medical records, extracting demographic and clinical data including age, sex, smoking status, implant survival or failure, implant location (maxilla or mandible; anterior or posterior), dental implant diameter and length, implant design (tapered or cylindrical), number of implants and type of implant-supported restoration (SC or FDP), bone type (pristine or regenerated), presence of simultaneous GBR, timing of implant placement (immediate vs. delayed), and duration of clinical follow-up. Pristine bone was defined as native bone without prior or simultaneous regenerative procedures, whereas regenerated bone included sites treated with sinus lift, alveolar ridge preservation, or guided bone regeneration. Smoking status was recorded as a binary variable (smoker vs. non-smoker), without further stratification according to the number of cigarettes per day. In the event of implant failure, the exact time (in months) was also noted, and whether or not it failed before loading.

Implant survival was defined as the absence of mobility, persistent infection, or progressive radiographic bone loss leading to implant removal [18,19]. Therefore, if any of these criteria appeared during follow-up for any of the O-PZIs, it was considered a failure. Biological or technical complications not resulting in implant failure were not systematically recorded due to the retrospective nature of the study.

### 2.4. Surgical Procedure

All surgeries were carried out by the same surgeon (J.C.-B.B.) under local anesthesia (4% articaine with 1:100,000 adrenalin). A full-thickness mucoperiosteal flap was elevated, both vestibular and palatal, by means of mid-crestal incision. When the alveolar bone had been exposed, the implant bed was prepared using a sequential drilling protocol with a round bur for cortical marking followed by pilot and twist drills of increasing diameter corresponding to the selected implant size, with depth-controlled preparation according to the manufacturer’s guidelines, prior to placement of O-PZIs.

All implants placed were Z-Systems^®^ implants (Oensingen, Switzerland), comprising cylindrical (Z5m^®^) and tapered (Z5m(t)^®^) designs, with a sandblasted, patented laser-modified micro-rough surface. When indicated, guided bone regeneration was performed with deproteinized bovine bone material (Bio-oss^®^, Geistlich^®^ (Wolhusen, Switzerland)) and a collagen membrane (Bio-gide^®^, Geistlich^®^ (Wolhusen, Switzerland). Due to the retrospective study design, defect morphology, size, and classification were not consistently recorded, and no standardized GBR protocol could be verified across all cases.

All patients were prescribed 1 g of amoxicillin 1 h before the surgical procedure [20] and an anti-inflammatory (400 mg Ibuprofen) every 6 h for 4 days in combination with 500 mg of acetaminophen every 8 h for 5 days for pain relief [21] and were advised to rinse with 0.2% chlorhexidine mouthwash every 12 h for 7 days. Sutures were removed after 7 days. In cases of simultaneous guided bone regeneration, the sutures were removed after 2 weeks.

The provisional restoration selected was a vacuum stent, serving both as provisional restoration and protective stent.

**Figure 1:**
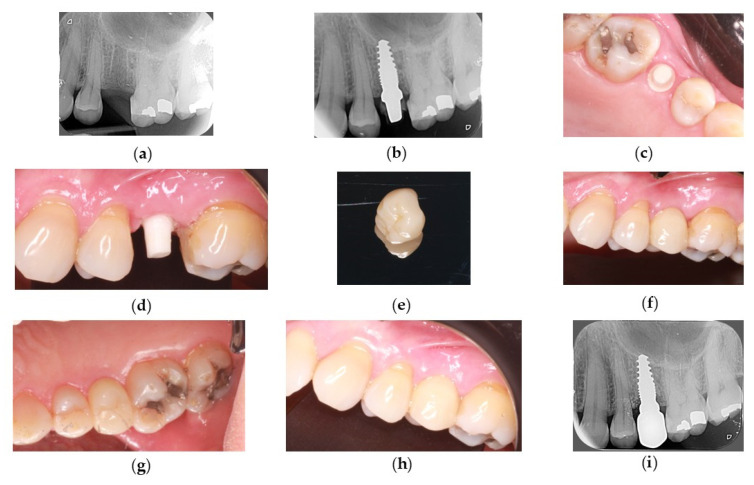
Complete clinical case with 3-year follow-up. (**a**,**b**) Periapical radiographs showing the post-extraction socket before implant placement and the tapered implant in place (Z-Systems Z5m(t) -4010). (**c**,**d**) Clinical images at the end of the osseointegration period. Note the health of the soft tissue. (**e**) Monolithic zirconium permanent crown manufactured using CAD/CAM technology. (**f**,**g**) Intraoral images of the crown in place. (**h**) Clinical image taken 3 years after placement. Note the excellent health of the soft tissue and integration of the restoration. (**i**) Periapical X-ray taken 3 years post-loading showing stable bone tissue.

After a 5-month osseointegration period in the maxilla or a 4-month period in the mandible, provisional prostheses were replaced with definitive restorations. At regenerated sites, the implants were left to heal for longer before receiving the definitive restoration. SCs or FDPs were fabricated from veneered or monolithic zirconia. The final prostheses were cemented onto the implant abutments with resin cement (RelyX Unicem 2, Aplicap, 3M, Espe). Any excess material was removed, and the restoration bonded, applying rigorous isolation measures to prevent cement from invading the areas adjoining the implant (Figure 1).

**Figure 2:**
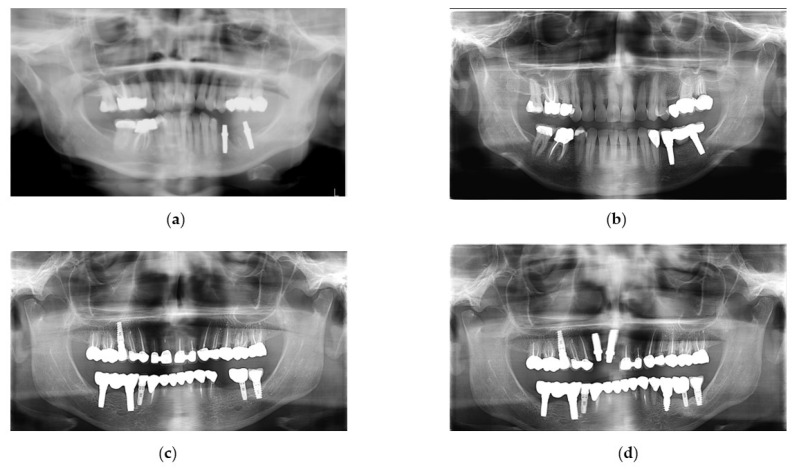
Panoramic radiographs of two patients: (**a**,**b**) 73-month follow-up of two O-PZIs supporting FDPs in the third quadrant; (**c**,**d**) 51-month follow-up of two O-PZIs supporting FDPs in the fourth quadrant and 23-month follow-up of one O-PZI supporting an SC in the third quadrant.

Follow-up visits were scheduled every 6–12 months after prosthesis delivery, followed by annual reviews. The minimum follow-up period was 39 months and the maximum was 96 months. These follow-up visits included professional maintenance (ultrasonic cleaning and supportive peri-implant and periodontal therapy tailored to individual needs) and clinical and radiographic examinations for peri-implant tissue health and prosthesis integrity according to the ALADA (As Low As Diagnostically Acceptable) principle and ADA/FDA guidelines [22] (Figure 2).

### 2.5. Statistical Analysis

Statistical analysis was conducted by an independent statistician. Data were analyzed with SPSS Statistics 29.0 software (SPSS^®^ Inc., Chicago, IL, USA).

Descriptive statistics (mean, median, standard deviation, percentage distribution, and confidence interval) were calculated for implant characteristics. Implant survival was analyzed using Kaplan–Meier survival curves. In addition, Cox regression was performed as a complement to survival analysis to assess risk factors, and a log-rank test was conducted to compare differences in survival between different groups. Statistical survival relationships were determined using the chi-square test. Statistical significance was established with a 95% confidence interval (CI) (*p* < 0.05, two-tailed).

Variables included in the model were selected based on clinical relevance and prior evidence drawn from the literature [23].

Analysis was conducted in terms of implant outcomes. Although a number of patients received various implants, no statistical adjustment for clustering was performed, which could have affected variance estimation. This limitation should be considered when interpreting the results.

## 3. Results

### 3.1. Participants

Data derived from 307 O-PZIs placed in 196 patients were analyzed. The sample comprised 204 women (66.45%. CI 95%: 61.03–71.56%) and 103 men (33.55%. CI 95%: 28.44–39.97%). The mean patient age at implant placement was 57.15 ± 11.14 years and the mean follow-up period was 61.37 ± 2.25 months (range: 39–96 months).

### 3.2. Descriptive Data

Of the 307 O-PZIs, 38 (12.38%. CI 95%: 9.05–16.41%) had a size of 3.6 × 10 mm; 22 (7.17%. CI 95%: 4.68–10.46%) had a size of 3.6 × 12 mm; 46 (14.98%. CI 95%: 11.33–19.30%) had a size of 4 × 8 mm; 127 (41.37%. CI 95%: 35.96–46.94%) had a size of 4 × 10 mm; 59 (19.22%. CI 95%: 15.11–23.90%) had a size of 4 × 12 mm; 1 (0.33%. CI 95%: 0.04–1.51%) had a size of 5 × 8 mm; 11 (3.58%. CI 95%: 1.92–6.12%) had a size of 5 × 10 mm; and 3 (0.98%. CI 95%: 0.28–2.58%) had a size of 5 × 12 mm. Regarding implant design, 87 implants (28.34%. CI 95%: 23.52–33.57%) were tapered, while 220 implants (71.66%. CI 95%: 66.43–76.48%) were cylindrical.

Concerning implant restoration, 148 implants (48.21%. CI 95%: 42.66–53.79% were restored with SCs and 159 (51.79%. CI 95%: 46.21–57.34%) with FDPs.

As for the distribution of implants (maxilla or mandible), 121 implants (39.41%. 95% CI: 34.07–44.96%) were placed in the maxilla and 186 implants (60.59%. 95% CI: 55.04–65.93%) in the mandible. Moreover, 46 implants (14.98%. 95% CI: 11.33–19.30%) were placed in the anterior region (incisors and canines) and 261 implants (85.02%. 95% CI: 80.70–88.67%) in the posterior region (premolars and molars).

Seventy implants (22.80%. CI 95%: 18.38–27.74%) were placed in patients who were smokers. A total of 242 implants (78.83%. CI 95%: 74.00–83.11%) were placed in pristine bone, while 65 implants (21.17%. CI 95%: 16.89–26.00%) were placed in regenerated bone (sinus lift, alveolar preservation, or some other GBR technique). Thirty-nine implants (12.70%. CI 95%: 9.33–16.77%) were placed simultaneously with guided bone regeneration and/or sinus lifting. Twenty-four implants (7.82%. CI 95%: 5.21–11.22%) were placed simultaneously with dental extraction.

The distribution of implants across these variables is summarized in Table 1. No missing data were identified for the variables included in survival and regression analyses.

### 3.3. Outcome Data

During the observation period, a total of 42 implant failures (13.68%. CI 95%: 10.00–18.14%) were recorded among the 307 O-PZIs. These 42 implants failed in 42 patients, resulting in an overall cumulative implant survival rate of 86.32% (CI 95%: 79.28–92.96%) at implant level and 78.57% (CI 95%: 62.29–84.61%) at patient level.

The failures occurred at different time points throughout the follow-up periods: 27 (out of 42) (64.29%. CI 95%: 56.77–71.73%) did so before loading (0–6 months), 8 (out of 42) (19.05%. CI 95%: 16.82–21.25%) failed between >6 and 24 months after placement and 7 (out of 42) (16.67%. CI 95%: 14.76–19.59%) failed between >24 and 60 months after placement. Characteristics of the failed implants are shown in Table 2.

### 3.4. Survival Analysis

#### 3.4.1. Bivariate Analysis

Bivariate chi-square analysis demonstrated a statistically significant association between implant survival and restoration type as well as simultaneous GBR (*p* < 0.05).

Implants supporting SCs showed a survival rate of 80.40% compared with 91.82% for implants supporting FDPs (*p* = 0.004). Similarly, implants placed with simultaneous GBR exhibited a lower survival rate (71.79%) compared with those placed without regeneration procedures (88.43%) (*p* = 0.005). Among the 11 implants that failed following simultaneous GBR, 2 failures were attributed to infection at the regenerated site, while 9 were due to insufficient stability.

No statistically significant associations were observed between implant survival and implant morphology, implant design, bone type, or smoking (*p* > 0.05). Detailed survival percentages and *p*-values are shown in Table 3.

#### 3.4.2. Kaplan–Meier Survival Analyses

Kaplan–Meier survival curves were constructed to evaluate time-to-event differences among clinical variables.

Survival analysis based on implant-supported restoration (SCs versus FDPs) revealed no statistically significant differences in implant survival; the log-rank test demonstrated no significant differences between survival curves (χ^2^ = 0.790. df = 1; *p* = 0.364).

**Figure 3:**
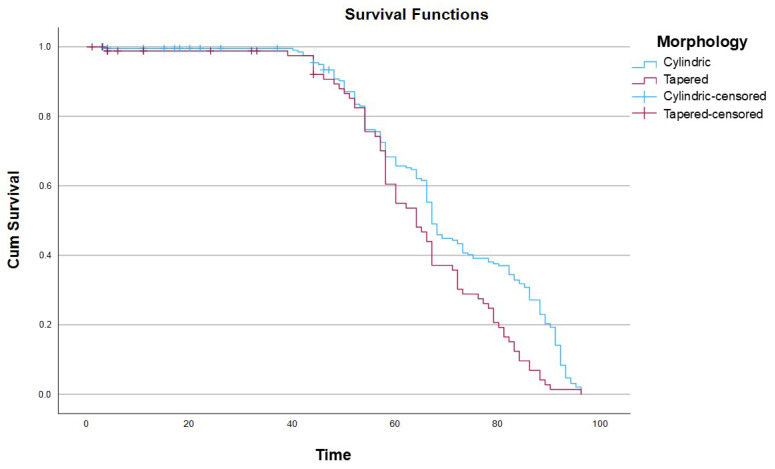
Kaplan–Meier survival curves at 96 months according to implant morphology.

When survival was analyzed according to implant morphology, a significantly lower cumulative survival rate was observed for tapered implants compared with cylindrical implants. This difference was statistically significant, as confirmed by the log-rank test (χ^2^ = 11.349. df = 1; *p* < 0.001) (Figure 3).

Survival analysis according to bone type showed no statistically significant differences between implants placed in pristine bone and those placed in regenerated bone. The log-rank test revealed no significant differences between survival curves (χ^2^ = 0.170. df = 1; *p* = 0.679).

**Figure 4:**
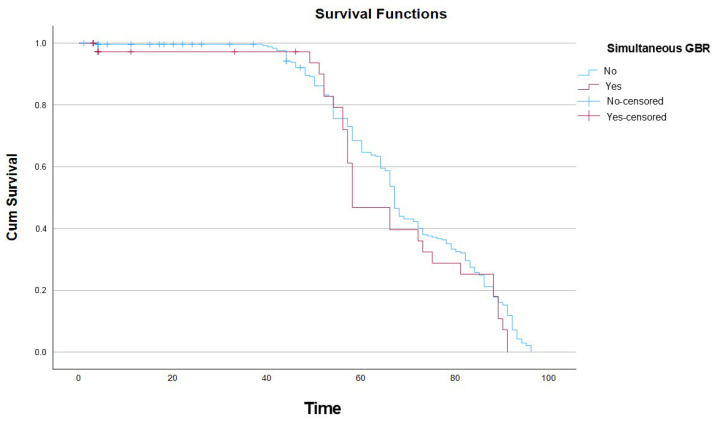
Kaplan–Meier 96-month survival curves according to simultaneous GBR.

Similarly, in univariate analysis, the use of simultaneous GBR at the time of implant placement did not result in statistically significant differences in implant survival. The log-rank test demonstrated no significant differences between the survival curves of implants placed with or without simultaneous GBR (χ^2^ = 1.670. df = 1; *p* = 0.196) (Figure 4).

No statistically significant differences in implant survival were observed when immediate implant placement was compared with delayed placement after tooth extraction. The log-rank test confirmed the absence of significant differences between survival curves (χ^2^ = 0.198. df = 1; *p* = 0.656).

**Figure 5:**
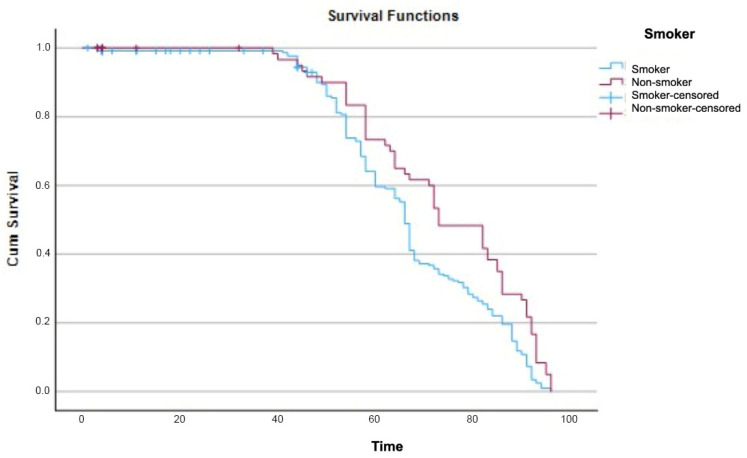
Kaplan–Meier 96-month survival curves according to smoking status.

In contrast, survival analysis based on smoking status demonstrated a significantly lower cumulative survival rate among smokers compared with non-smokers. The log-rank test confirmed a statistically significant difference between the survival curves (χ^2^ = 10.116. df = 1; *p* < 0.001) (Figure 5).

#### 3.4.3. Multivariate Analysis

The Cox regression method was used to analyze simultaneous GBR. The results presented lower odds of survival in implants placed simultaneously with GBR compared to those placed without GBR (Exp(B) = 3.191. CI 95%: 1.299–7.840%) with statistically significant difference (*p* = 0.011). In other words, the probability of implant failure increased by 3.191 times when the O-PZIs were placed simultaneously with GBR.

Other variables, including implant morphology, smoking status, implant design, bone type and timing of placement, did not reach statistical significance in the multivariate model (*p* > 0.05). The results of the multivariate analysis are shown in Table 4.

## 4. Discussion

This retrospective cohort study evaluated the medium- to long-term clinical survival of O-PZIs and explored potential factors associated with implant failure. The overall cumulative implant survival rate was 86.32% (CI 95%: 79.28–92.96%) at implant level and 78.57% (CI 95%: 62.29–84.61%) at patient level after a mean follow-up of 61.37 ± 2.25 months. These findings provide relevant information about a series of potential factors associated with implant failure.

The survival rate observed in the present study was lower than those reported in two recent SRs that ranged from 94.40% for O-PZIs to 96.31% for two-piece implants over shorter follow-up periods of 1–3 years [5,24]. However, other studies with follow-up periods of up to 15 years have obtained survival rates ranging between 59.26% and 98.69% [25,26,27,28,29], which align more closely with the present results. This supports the well-established concept that implant survival decreases over time, highlighting the importance of long-term observation when evaluating ceramic implant systems [30].

The overall survival rate of titanium implants is higher compared with zirconium implants. Jung et al. [31] observed a survival rate of 95.2% over 10 years for titanium implants supporting SCs, while Pjetursson et al. [32] obtained a survival rate of 93.1% over 10 years for implants supporting FDPs.

It should be noted that one-piece implants are immediately exposed to forces exerted by the tongue or resulting from chewing [33,34,35]. At the same time, the initial healing period depends not only on the clinician’s decision but on patient compliance as well. In the present study, bivariate analysis showed that both the type of restoration and simultaneous GBR were significantly associated with implant survival. Implants supporting FDPs demonstrated higher survival rates compared with SCs, while implants placed with simultaneous GBR showed lower survival rates. However, when time-to-event analysis was performed using Kaplan–Meier curves, restoration type and simultaneous GBR no longer showed statistically significant differences, whereas implant morphology and smoking status did exhibit significant differences in survival curves. These discrepancies might be explained by differences between statistical methods. Bivariate analyses evaluate crude proportions without considering the temporal distribution of events, whereas Kaplan–Meier methods incorporate time-to-event data, and Cox regression further adjusts for the simultaneous influence of multiple covariates. Consequently, variables identified as significant in unadjusted analyses may lose statistical significance after accounting for time and confounding effects [36,37,38].

In the present study, in Multivariate Cox regression, only simultaneous GBR remained independently associated with implant failure (Exp(B) = 3.191. 95% CI: 1.299–7.840; *p* = 0.011). The odds (probability of implant failure) increased by 3.191 times when the O-PZIs were placed simultaneously with GBR. However, this finding should be interpreted with caution and should be limited only to the variables included in this model. The retrospective design, the limited number of failure events, and the potential influence of unmeasured confounders may have affected the stability of the model. Furthermore, the analysis was conducted in terms of implants without accounting for clustering within patients, which may have influenced variance estimation and *p*-values. Additionally, the relatively limited number of failure events in relation to the number of variables included in the Cox regression model may have affected model stability. Although variables were selected based on clinical relevance and prior evidence, the events-per-variable ratio was relatively low, which may increase the risk of overfitting and reduce the robustness of the results. Therefore, the association observed should be considered exploratory rather than definitive.

The association between simultaneous GBR and lower implant survival may reflect the biological complexity of augmented sites. Reduced vascularization alters bone remodeling; this can make it difficult to achieve optimal primary stability and could contribute to early implant failure [39,40]. However, due to the lack of standardized reporting of defect characteristics and failure modes, it was not possible to determine the underlying mechanisms in this cohort. In this context, it should be noted that Kaplan–Meier analysis did not reveal significant differences between pristine and regenerated bone, suggesting that the timing of regenerative procedures may play a more relevant role than the mere presence of regenerated bone. Moreover, given their clinical interrelationship, the potential collinearity between bone type and simultaneous GBR was considered during model construction. Both variables were retained due to their distinct clinical meaning (baseline bone condition vs. timing of regeneration), although some degree of overlap cannot be discounted. This finding should be interpreted with caution and warrants further investigation.

A high proportion of failures occurred before prosthetic loading, which may be partially related to the one-piece implant design. Immediate transmucosal exposure may increase susceptibility to unintended micro-loading during healing, potentially compromising osseointegration, particularly in situations with suboptimal primary stability. This aspect should be considered when interpreting early biological failures in one-piece implant systems [41].

Regarding implant morphology, Kaplan–Meier analysis demonstrated significantly lower cumulative survival for tapered implants compared with cylindrical designs. A possible explanation may be the higher insertion torque (<50 Ncm) frequently required for tapered implants, potentially leading to excessive compression of the cortical bone, local ischemia, and early biological complications [42]. Nevertheless, this variable did not remain significant in the multivariate model, suggesting that its effect may be influenced by other factors such as bone condition or case selection.

Likewise, smoking was significantly associated with lower survival in Kaplan–Meier analysis, consistent with extensive evidence demonstrating that tobacco use negatively affects osseointegration due to impaired vascularization, altered immune response, and delayed bone healing [43,44]. Similar findings have been reported in other studies of ceramic implants [45,46]. However, this variable did not remain significant in the multivariate model, which may be related to limited statistical power, lack of stratification of smoking intensity, or interaction with other clinical variables. Similarly, implant morphology showed an association in univariate survival analysis but not after adjustment, suggesting that its effect may be influenced by other factors such as bone conditions or surgical variables.

It is important to emphasize that implant failure is a multifactorial process influenced not only by surgical and prosthetic variables but also by host-related factors, including systemic conditions, immune response, and potential genetic predisposition. Moreover, implant failure is a biologically complex process involving dynamic interaction between biomaterials and host tissues. In this context, the host immune response plays a critical role in modulating osseointegration, as an excessive or dysregulated inflammatory reaction may impair early healing and compromise bone formation. Furthermore, bone metabolism and remodeling dynamics are essential for implant stability, particularly during the early phases of healing, where a balance between osteoblastic activity and bone resorption is required. In augmented sites, these processes may be altered due to reduced vascularization, delayed maturation of regenerated bone, and changes in the local biological environment, potentially increasing susceptibility to early implant failure [47,48]. These variables were not available in the present dataset and should be considered when interpreting the results. Consequently, the associations identified are limited to the variables included in the analysis.

This study has several limitations. Its retrospective design introduces potential selection and attrition bias and limits control over confounding variables. Relevant factors such as periodontal status, oral hygiene, occlusal loading, insertion torque, the type of implant failure and primary stability were not consistently recorded. Biological and technical complications not leading to implant failure were not systematically recorded, and failure modes could not be classified consistently. The absence of adjustment for clustering in terms of patients receiving more than one implant may have affected statistical inference. In addition, the relatively low number of failure events in relation to the number of variables included in the multivariate model may have increased the risk of overfitting. Moreover, although loss to follow-up was clearly documented, the potential impact of attrition bias should be considered. Patients who did not complete follow-up may have experienced different clinical outcomes compared with those retained in the study, which could have influenced the estimated survival rates. Finally, the external validity of the present findings may be limited. The study was conducted in a single private clinical setting with specific inclusion criteria and a standardized surgical approach, which may not fully reflect broader clinical practice conditions. Therefore, caution should be exercised when extrapolating these results to different populations or clinical environments. These limitations should not be interpreted in isolation but rather in direct relation to the study findings. In particular, the absence of key host-related variables—such as periodontal status, oral hygiene, occlusal loading, and systemic or genetic factors—may have introduced unmeasured confounding, potentially influencing the observed associations. Consequently, the identification of independent predictors in the present model should be interpreted with caution, as these factors may partially account for the variability in implant outcomes.

Despite these limitations, this study provides one of the largest retrospective cohorts with medium- to long-term follow-up for O-PZIs published to date and contributes clinically relevant data on their performance in routine practice.

Future prospective, controlled comparative studies with standardized surgical protocols together with assessment of other valuable clinical variables such as marginal bone loss (MBL) or biological parameters such as Probing Depth (PD), Bleeding Index (BI) and Plaque Index (PI) are necessary to confirm the present findings and further clarify the influence of regenerative procedures and implant design on long-term survival.

## 5. Conclusions

Within the limitations of this retrospective cohort study, one-piece zirconia implants demonstrated a cumulative survival rate of 86.32% (CI 95%: 79.28–92.96%) over a mean follow-up of 61.37 ± 2.25 months.

Although several variables showed associations with implant survival in unadjusted analyses, multivariate Cox regression identified simultaneous guided bone regeneration as the only variable significantly associated with implant failure. However, this finding should be interpreted with caution due to methodological limitations, including potential confounding, limited event numbers, and lack of adjustment for clustered data.

The discrepancies observed between bivariate, Kaplan–Meier, and multivariate analyses underscore the importance of using appropriate time-to-event statistical methods when evaluating implant outcomes.

From a clinical perspective, careful case selection—particularly in sites requiring simultaneous regenerative procedures—may be relevant when planning treatment with one-piece ceramic implants. Nevertheless, the present findings should be considered exploratory, and further well-designed prospective studies are needed to confirm these associations and to refine risk assessment strategies.

## Figures and Tables

**Table 1:** Distribution of implant characteristics.

Implant Characteristics		No. of Implants	Percentage (%)
Type of restoration	SC	148	48.21 (42.66–53.79)
	FDP	159	51.79 (46.21–57.34)
Morphology	Tapered	87	28.34 (23.52–33.57)
	Cylindrical	220	71.66 (66.43–76.48)
Bone type	Pristine	242	78.83 (74.00–83.11)
	Regenerated	65	21.17 (16.89–26.00)
Simultaneous GBR	Yes	39	12.70 (9.33–16.77)
	No	268	87.30 (83.23–90.67)
Post-extraction implant	Yes	24	7.82 (5.21–11.22)
	No	283	92.18 (88.78–94.79)
Smoking	Yes	70	22.80 (18.38–27.74)
	No	237	77.20 (72.26–81.62)

(SC: single crown; FDP: fixed dental prostheses; GBR: guided bone regeneration).

**Table 2:** Details of failed implants.

No.	Gender	Site	Diameter and Length (mm)	Shape	Smoker Habit	Bone Type	Type of Restoration	Simultaneous Grafting	Post-Extraction	Moment of Failure (Months)
1	Man	Mb	4 × 12	Tapered	No	Pristine bone	SC	No	No	24
2	Man	Mx	4 × 10	Cylindrical	Yes	Pristine bone	SC	Yes	No	Before loading
3	Woman	Mx	4 × 8	Cylindrical	No	Pristine bone	FDP	Yes	No	Before loading
4	Man	Mb	4 × 12	Tapered	No	Pristine bone	SC	Yes	No	Before loading
5	Woman	Mb	4 × 8	Cylindrical	Yes	Pristine bone	FDP	No	No	Before loading
6	Woman	Mx	3.6 × 10	Cylindrical	No	Pristine bone	FDP	Yes	No	Before loading
7	Woman	Mb	5 × 10	Cylindrical	Yes	Pristine bone	SC	No	No	Before loading
8	Man	Mb	4 × 10	Cylindrical	No	Pristine bone	FDP	Yes	Yes	Before loading
9	Woman	Mx	4 × 10	Cylindrical	No	Pristine bone	SC	No	No	Before loading
10	Man	Mx	3.6 × 10	Cylindrical	No	Regenerated bone	SC	No	No	Before loading
11	Woman	Mb	5 × 10	Cylindrical	Yes	Regenerated bone	SC	No	No	Before loading
12	Man	Mx	4 × 12	Tapered	No	Regenerated bone	SC	Yes	No	19
13	Woman	Mx	4 × 10	Cylindrical	No	Regenerated bone	FDP	No	No	Before loading
14	Man	Mb	4 × 10	Cylindrical	No	Regenerated bone	SC	No	No	Before loading
15	Man	Mx	3.6 × 12	Cylindrical	Yes	Pristine bone	FDP	No	No	Before loading
16	Woman	Mx	4 × 10	Cylindrical	No	Pristine bone	SC	No	No	Before loading
17	Man	Mb	4 × 10	Cylindrical	No	Pristine bone	FDP	No	No	Before loading
18	Woman	Mb	4 × 10	Cylindrical	No	Pristine bone	SC	Yes	No	Before loading
19	Man	Mb	4 × 10	Cylindrical	No	Regenerated bone	FDP	No	No	Before loading
20	Woman	Mx	4 × 8	Cylindrical	Yes	Regenerated bone	FDP	No	No	Before loading
21	Woman	Mb	5 × 10	Cylindrical	No	Pristine bone	SC	No	No	32
22	Woman	Mx	4 × 10	Tapered	No	Regenerated bone	SC	No	No	30
23	Man	Mx	4 × 12	Tapered	No	Pristine bone	SC	Yes	Yes	Before loading
24	Woman	Mb	4 × 10	Tapered	No	Pristine bone	SC	No	No	Before loading
25	Man	Mb	4 × 10	Cylindrical	No	Regenerated bone	FDP	No	No	Before loading
26	Woman	Mb	4 × 10	Cylindrical	No	Pristine bone	FDP	No	No	19
27	Woman	Mx	4 × 8	Cylindrical	Yes	Regenerated bone	FDP	Yes	No	Before loading
28	Woman	Mb	5 × 10	Cylindrical	Yes	Pristine bone	SC	No	No	11
29	Woman	Mx	4 × 10	Tapered	No	Pristine bone	SC	No	No	11
30	Man	Mx	4 × 12	Tapered	No	Regenerated bone	SC	Yes	Yes	Before loading
31	Man	Mx	4 × 8	Tapered	Yes	Pristine bone	SC	No	No	32
32	Woman	Mx	4 × 10	Tapered	No	Pristine bone	SC	No	Yes	44
33	Man	Mx	4 × 12	Tapered	Yes	Pristine bone	SC	No	No	Before loading
34	Man	Mb	4 × 10	Cylindrical	No	Pristine bone	SC	No	No	47
35	Man	Mb	4 × 10	Cylindrical	No	Pristine bone	SC	No	No	18
36	Woman	Mx	3.6 × 10	Cylindrical	No	Pristine bone	SC	No	No	Before loading
37	Woman	Mb	4 × 10	Tapered	No	Pristine bone	SC	No	No	Before loading
38	Woman	Mb	4 × 10	Tapered	No	Pristine bone	SC	No	No	17
39	Woman	Mx	3.6 × 12	Tapered	No	Regenerated bone	SC	Yes	Yes	Before loading
40	Man	Mb	4 × 8	Cylindrical	No	Pristine Bone	SC	No	No	44
41	Woman	Mx	4 × 10	Cylindrical	No	Pristine bone	SC	No	No	22
42	Woman	Mx	4 × 10	Cylindrical	No	Pristine bone	FDP	No	No	26

(Mb: mandible; Mx: maxilla; SC: single crown; FDP: fixed dental prostheses.)

**Table 3:** Implant-based survival rate comparison.

Implant Characteristics	Variables	Failed Implants/Total Number of Implants	Success/Survival Rate (%)	*p*-Value
Type of restoration	SC FDP	29/148 13/159	80.40 91.82	0.004
Design	Cylindrical Tapered	28/220 14/87	87.27 83.90	0.439
Bone type	Pristine bone Regenerated bone	30/242 12/65	87.60 81.54	0.206
Simultaneous GBR	Yes No	11/39 31/268	71.79 88.43	0.005
Immediate implants	Yes No	5/24 37/283	79.16 86.92	0.288
Smoking habit	Yes No	10/70 32/237	85.71 86.50	0.867

(SC: single crown; FDP: fixed dental prostheses; GBR: guided bone regeneration).

**Table 4:** Results of multivariate analysis.

Variable	Category	Exp(B)	95% CI	*p*-Value
Restoration type	SC vs. FDP	0.421	0.229–2.763	0.298
Implant morphology	Tapered vs. Cylindrical	0.674	0.434–3.115	0.108
Smoking status	Smoker vs. Non-smoker	0.853	0.319–2.142	0.074
Bone type	Pristine vs. Regenerated	0.609	0.289–1.286	0.193
Simultaneous GBR	No vs. Yes	3.191	1.299–7.840	0.011
Timing of placement	Immediate vs. Delayed	0.870	0.260–2.914	0.822

(GBR: guided bone regeneration; SC: single crown; FDP: fixed dental prostheses).

## Data Availability

The original contributions presented in this study are included in the article. Further inquiries can be directed to the corresponding author.

## Abbreviations

The following abbreviations are used in this manuscript:

O-PZI	One-Piece Zirconia Implants
GBR	Guided Bone Regeneration
SR	Systematic Review
SC	Single Crown
FDP	Fixed Dental Protheses
Mx	Maxilla
Mb	Mandible
ALADA	As Low As Diagnostically Acceptable
MBL	Marginal Bone Loss
PI	Plaque Index
PD	Probing Depth
BI	Bleeding index
Y-TZP	Yttria-Stabilized Tetragonal Zirconia Polycrystal
ATZ	Alumina-Toughened Zirconia
